# Composite Metal Oxide Nanopowder-Based Fiber-Optic Fabry–Perot Interferometer for Protein Biomarker Detection

**DOI:** 10.3390/bios15070449

**Published:** 2025-07-13

**Authors:** Ulpan Balgimbayeva, Zhanar Kalkozova, Kuanysh Seitkamal, Daniele Tosi, Khabibulla Abdullin, Wilfried Blanc

**Affiliations:** 1 School of Materials Science and Green Technology, Kazakh-British Technical University, Almaty 050000, Kazakhstan; u.balgimbaeva@kbtu.kz; 2 National Nanotechnology Laboratory of Open Type, Al-Farabi Kazakh National University, Almaty 050040, Kazakhstan; zhanar.kalkozova@kaznu.edu.kz; 3 Laboratory of Biosensors and Bioinstruments, National Laboratory Astana, Nazarbayev University, Astana 010000, Kazakhstan; kuanysh.seitkamal@nu.edu.kz; 4 School of Engineering and Digital Sciences, Nazarbayev University, Astana 010000, Kazakhstan; 5 INPHYNI, CNRS UMR7010, Université Côte d’Azur, 17 rue Julien Lauprêtre, 06200 Nice, France; wilfried.blanc@inphyni.cnrs.fr

**Keywords:** nanopowder, optical fiber biosensors, single-mode fibers, Fabry–Perot interferometry, biomarker detection

## Abstract

In this paper, we present the development of a new semi-distributed interferometer (SDI) biosensor with a Zn, Cu, and Co metal oxide nanopowder coating for the detection of a kidney disease biomarker as a model system. The combination of nanopowder coating with the SDI platform opens up unique opportunities for improving measurement reproducibility while maintaining high sensitivity. The fabrication of sensors is simple, which involves one splice and subsequent cutting at the end of an optical fiber. To ensure specific detection of the biomarker, a monoclonal antibody was immobilized on the surface of the probe. The biosensor has demonstrated an impressive ability to detect biomarkers in a wide range of concentrations, from 1 aM to 100 nM. The theoretical limit of detection was 126 fM, and the attomolar detection level was experimentally achieved. The sensors have achieved a maximum sensitivity of 190 dB/RIU and operate with improved stability and reduced dispersion. Quantitative analysis revealed that the sensor’s response gradually increases with increasing concentration. The signal varies from 0.05 dB at 1 aM to 0.81 dB at 100 nM, and the linear correlation coefficient was R^2^ = 0.96. The sensor showed excellent specificity and reproducibility, maintaining detection accuracy at about 10^−4^ RIU. This opens up new horizons for reliable and highly sensitive biomarker detection, which can be useful for early disease diagnosis and monitoring using a cost-effective and reproducible sensor system.

## 1. Introduction

The development of new technologies and approaches that significantly increase the sensitivity of optical fiber-based sensors has been the subject of several studies over the last decade [1]. Interferometric methods and refractive index (RI) measurements deserve special attention due to their high sensitivity and precision [2]. One of the key optical characteristics of any material that establishes its electromagnetic characteristics and influences the speed at which light propagates in phase space is its RI [3]. Highly sensitive sensors capable of detecting the slightest changes in RI are especially important in biosensor applications. They make it possible to identify biomarkers with high accuracy [4,5].

Fiber-optic (FO) technologies represent an attractive platform for measuring RI due to their high sensitivity, compactness, and ability to integrate. In such systems, both internal and external probes can be used, which register changes in RI in the environment [6]. One of the most common methods in FO sensors is interferometry, which is highly accurate and capable of detecting changes at the 10^−6^ refractive index unit (RIU) level [7,8]. What’s more, FO sensors use different interferometric configurations such as Mach-Zehnder, Michelson, or Fabry–Perot (F-P), where these configurations convert changes in the RI into phase shifts and spectral variations that occur due to changes in the optical path difference between the branches of the interferometer. In alternative schemes, the fiber probe can act as a reflective element [9]. Its optical properties vary depending on the environment, which opens up additional possibilities for measuring the RI. Alternatively, the tip of the fiber can be used as a mirror, the reflectivity of which depends on the RI. This causes significant changes in the reflection or transmission spectra. In the case of label-free biosensors, the surface of the sensors is subjected to special treatment [10], such as silanization or coating with nanomaterials. As a result, platforms are being created on which bioreceptors capable of detecting certain biomolecules can be integrated. These biosensors operate on the principle of interferometry and are highly sensitive. They can measure phase changes that occur when biomolecules interact, which, in turn, is associated with changes in the RI.

It has been reported that single-mode fibers (SMFs) equipped with semi-distributed interferometers (SDIs) are successfully used to detect protein biomarkers [10,11]. In addition, F-P optical fiber sensors, which operate based on the principles of optical interference, have become widely used to determine biochemical parameters [12,13]. These F-P sensors combine the advantages of fiber-optic devices, such as compactness and resistance to electromagnetic interference, with practical advantages, including ease of production, cost-effectiveness, and the possibility of probe-based configuration.

Combining the interferometric detection method with nanoparticle-enhanced sensor surfaces opens up new horizons. Using various strategies for functionalizing, the surface of nanoparticles, sensitive biomolecules such as proteins, nucleic acids, peptides, and small molecules can be attached to their surface. This makes it possible to create biosensors without tags that respond to changes in RI in their immediate vicinity [14]. These devices, combining cutting-edge materials science and sensors, open up new horizons in the field of biomarker detection. Coatings made of nanomaterials of complex metal oxides, such as Cu [15], Zn [16], and Co [17], are used in biosensor technologies in the interferometer design. For instance, the main advantages of cobalt Co MNP [17] coatings are their fast response in detecting the test substance, detection efficiency due to the significant linear range, and the structure of the synthesized nanoparticle. These materials are especially effectively combined with surface modification methods, which makes it possible to achieve maximum RI sensitivity.

Nanomaterials in biosensors can be used in different structures. For example, magnetic cobalt oxide nanoparticles can be an effective coating for an optical fiber sensor, significantly increasing the linear range and reducing the response time for biomarker concentration detection [18]. Nanopowders based on copper [15], zinc [16], and cobalt metal oxides [17] are used as coatings in biosensing applications due to their excellent electrical conductivity, optical properties, high surface area, and stability. Nanopowders based on copper, zinc, and cobalt oxide metals are promising materials for the use of various types of biosensors due to the simple and environmentally friendly nature of the material [17] and the low-cost hydrothermal synthesis method. The main distinctive advantages of metal oxide nanomaterials are good electric field [19], selectivity, sensitivity, stability, active surface area, and interaction with other substances in an alkaline environment, without deforming the basic parameters and functions of biosensors [20]. Applying these nanomaterials to optical fiber sensors is carried out in a real environment [19], with intensive interaction of the sensor surface with nanomaterials in the form of solution. The obtained sensors with modification improve their properties and characteristics and create favorable conditions for protein detection and other biomarkers. Table 1 shows a comparative analysis of composite metal oxide nanomaterials used as coatings to improve sensor properties. Thus, it should be noted that the characterization of the developed materials is in line with current technologies and other sensors used in similar applications.

In this study, we introduce an SMF-based label-free optical F-P interferometric biosensor coated with Zn, Cu, and Co metal oxide nanopowders for the selective and sensitive detection of a model protein biomarker—kidney injury molecule 1 (KIM-1), a biomarker of acute kidney injury and chronic kidney disease. Metal oxide nanostructures were synthesized and applied to the sensors using a dip-coating technique in order to enhance the surface available for biomolecular interactions, thereby improving sensitivity. While the same sensor platform has been previously employed for KIM-1 detection [21], in this study, we utilize metal oxide nanopowders to enhance performance. To build a biosensor, the fabricated and calibrated sensors were functionalized with antibodies specific to KIM-1 using silanization, enhancing its surface for biomolecular interactions.

**Table 1:** Comparison of the developed composite metal oxide nanomaterials with other types of sensors.

Nanomaterials	Sensor Type	Limit of Detection, LoD	Detection Range	References
Co_6_(CO_3_)_2_ (OH)_8_ H_2_O	Enzyme-free glucose sensors	16 µM	0.2–2 mM	[22]
ZnO/Co_3_O_4_	Non-enzymatic electrochemical amperometry glucose sensor	0.043 μM	0.015–10 mM	[23]
Co_3_O_4_/SWCNT	Non-enzymatic electrochemical amperometry glucose sensor	0.25 μM	1–5 mM	[24]
ZnO nanowires	Optical fiber plasmonic biosensors	0.51 pg/mL	0.01 pg/mL–1 ng/mL	[25]
Zn_(1−x)_CuCo_2_O_4_	Optical fiber biosensors	126 fM	1 aM to 100 nM	This study

## 2. Materials and Methods

### 2.1. Chemicals

Zinc nitrate hexahydrate (Zn(NO_3_)_2_·6H_2_O), cobalt nitrate hexahydrate (Co(NO_3_)_2_·6H_2_O), silicon nitrate hexahydrate (Si(NO_3_)_2_·6H_2_O), and urea (CH_4_N_2_O) were purchased from Sigma-Aldrich (St. Louis, MO, USA), while nickel foam, acetone, and ethanol were obtained from local suppliers. Distilled water (18.2 MΩ·cm), obtained using an ARIUM 611 DI water purification system (Sartorius Group), was used for the synthesis of nanopowders.

### 2.2. Hydrothermal Synthesis Process of the Nanopowders for Biosensors

**Figure 1:**
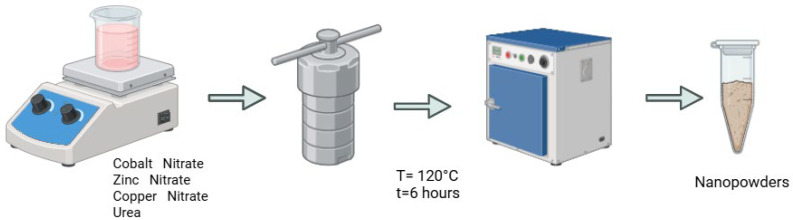
The process of synthesizing nanopowders for biosensing purposes. Created in BioRender. Balgimbayeva, U. (2025) https://BioRender.com/hucu8eb (accessed on 21 April 2025).

Zn_(1−x)_CuCo_2_O_4_ on nickel foam was synthesized using the hydrothermal method. A 2 cm by 1 cm piece of nickel foam was first purified through multiple stages. Initially, it was treated with 3% hydrochloric acid in an ultrasonic bath for 20 min, followed by multiple rinses in distilled water [26]. The foam was then immersed in acetone for 10 min and in ethanol for 10 min and washed again in distilled water before being dried at 80 °C in a drying oven. For the precursor solution, a mixture of zinc nitrate, cobalt nitrate, copper nitrate, urea (in varying molar concentrations), and distilled water was prepared. Hydrothermal synthesis took place in an autoclave, where the nitrates were dissolved in 10 mL of distilled water per solution, with nickel foam placed vertically. The sealed autoclave was then placed in a muffle furnace preheated to 140 °C [26]. The synthesis temperature was set at 120 °C for 6 h. Afterward, the autoclave was cooled to room temperature. The treated nickel foam was placed in an ultrasonic bath, washed several times with distilled water to remove any remaining powders, and then processed in a centrifuge. Figure 1 shows the experimental setup used for the synthesis of Zn, Cu, and Co composite metal oxide nanopowders.

Three samples with different molar ratios of cobalt, zinc, and copper precursors were synthesized. For sample No. 1, the molar ratio of Co:Zn:Cu precursors was 4:1.8:0.2. For samples No. 2 and No. 3, the molar ratio of precursors was 4:0.2:1.8 and 4:1:1, respectively. The molar concentration of urea was always twice the molar concentration of cobalt. This rinsing procedure was repeated 2–3 times. Finally, the collected powders were dried in an oven for 24 h.

### 2.3. Characterization Techniques

Copper, zinc, and cobalt metal oxide nanopowders were characterized using different analytical techniques. X-Ray diffraction (XRD) analysis was performed on a MiniFlex X-ray diffractometer (Rigaku, Tokyo, Japan) using CuKα radiation at a wavelength of 1.5418 Å. Raman spectroscopy studies were performed on a Ntegra Spectra spectrometer (NT-MDT, Apeldoorn, Nertherlands) under excitation at 473 nm. Morphology and microstructure of the nanomaterials were studied using a scanning electron microscope (SEM), Quanta 200 microscope (FEI, Hillsboro, OR, USA). In order to analyze the heat resistance and reliability of the samples, they were analyzed after annealing at 200 °C for 5 h in air using the same characterization techniques.

### 2.4. Fabrication of Interferometer-Based Sensors

**Figure 2:**
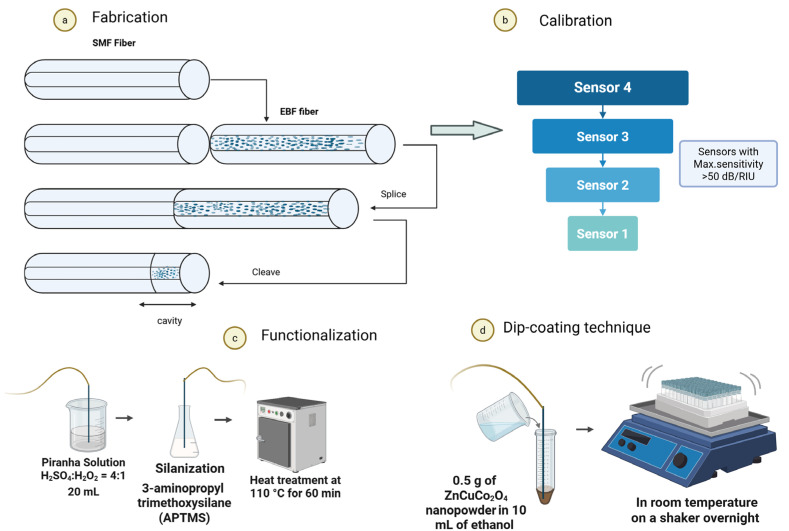
The process of sensor preparation for coating with nanopowders. (**a**) Schematic representation of an SDI sensor, (**b**) selection of sensors sensitive to changes in refractive index during calibration, (**c**) functionalization of sensors, and (**d**) ZnCuCo_2_O_4_ (No. 1 sample) nanopowder dip-coating technique on sensors. Created in BioRender. Balgimbayeva, U. (2025) https://BioRender.com/8ny4x95 (accessed on 21 April 2025).

The biosensors created in this study utilized SDI sensors as the sensing component, and sensors were manufactured based on the methods outlined in the previous research, which can be called a “splice-and-cleave” method. Manufacturing a reflecting probe sensitive to RI change only needs two operations: (1) splicing two fibers and (2) cleaving at the area close to the spliced region to create a mirror at the tip. Initially, to fabricate an SDI sensor, a conventional single-mode fiber (SMF-28, Corning, NY, USA) was used. It was connected to an EBF, which acts as the reflective element. The splicing was performed using a standard telecommunication fiber splicer (Fujikura 12-S, Tokyo, Japan) using the SMF-SMF protocol. The input SMF has a core with a diameter of 8.2 μm and a cladding with a diameter of 125 μm with corresponding refractive indices of 1.4682 and 1.4628, respectively. These values were the same for all fiber segments used in the sensors. While the EBF used in this study is based on the standard SMF structure, it has the addition of MgSiO_3_ nanoparticles to the core. These nanoparticles enhance backscattering but do not significantly change the bulk refractive index of the core, which remains approximately equal to 1.464. Figure 2 shows the algorithm of sensor preparation for nanopowder coating by the dip-coating method.

### 2.5. Calibration of Sensors

To assess the sensor’s responsiveness to RI changes, calibration was performed. Following production, each sensor underwent calibration using six distinct sucrose solutions with RI values ranging from 1.3476 to 1.3585. The lowest RI corresponded to a 10% sucrose solution. Other samples were prepared by adding 400 μL of 40% sucrose to a plastic vial containing 6 mL of 10% sucrose at each step. All solutions were prepared and used at room temperature (~25 °C), under which the solubility limit of sucrose is approximately 200 g per 100 mL (68% *w*/*v*). The highest sucrose concentration used in this calibration (approximately 13.9%) was well below the solubility limit, ensuring complete dissolution and avoiding crystallization or instability. This concentration range was selected to generate refractive indices relevant to typical biological and diagnostic fluids. The RI values were confirmed using a digital refractometer (Abbemat refractometer 3000, Anton Paar, Ashland, VA, USA) to ensure accurate sensor calibration across the intended detection range. Linear regression was used to analyze RI sensitivity, which was determined for peaks and valleys with R^2^ > 0.90. The sensor was linked to the interrogator, incorporating a fiber Bragg grating (FBG) for temperature control and to enhance spectral detection [27]. Sensors coated with the nanopowders were calibrated in a similar manner. Following calibration, comparative analysis between the sensors before and after nanoparticle modification was performed.

### 2.6. Optical Fiber Surface Coating with the ZnCuCo_2_O_4_ Nanopowder Thin Layer

Before coating with ZnCuCo_2_O_4_ (No. 1 sample), the surface of the optical fibers was cleaned in 20 mL of Piranha solution (H_2_SO_4_:H_2_O_2_ = 4:1) for 15 min to remove organic impurities and increase -OH groups. Then, the sensors were washed with distilled water and dried with nitrogen gas, followed by silanization in a 1% solution of (3-aminopropyl) trimethoxysilane (APTMS) in methanol solution for 30 min. Next, the optical fibers were heat-treated at 110 °C for 60 min and then coated with a layer of ZnCuCo_2_O_4_ (No. 1 sample) using the dip-coating technique. The nanopowder was deposited by dissolving 0.5 g of ZnCuCo_2_O_4_ (No. 1 sample) in 10 mL of ethanol at room temperature on a shaker overnight. Afterwards, the solution was uniformly applied to the surface of the fibers by dip-coating technique, and then the sensors were washed with distilled water and were dried in a muffle furnace at 110 °C for 10 min.

### 2.7. Biofunctionalization of Sensors

The modification of SDI sensors involves a series of steps to prepare fully functionalized sensors. After calibration of optical fiber sensors with nanopowders, they were washed again in PBS solution and were immersed in a 95% MUA (11-Mercaptoundecanoic acid) solution prepared in ethanol and incubated at −4 °C for 16 h to optimize cross-linking. The fibers were then washed with ethanol, incubated in an EDC (1-Ethyl-3-(3-dimethylaminopropyl) carbodiimide)/NHS (N-Hydroxysuccinimide) solution for 15 min, and rinsed with ethanol [28]. For antibody immobilization, the fibers were incubated in 500 µL of anti-KIM-1 monoclonal antibody solution (2 µg/mL) overnight at 4 °C. To block any unreacted carboxyl groups, the fibers were treated with 1% bovine serum albumin (BSA) for one hour and then rinsed with PBS before protein detection.

### 2.8. Protein Detection

**Figure 3:**
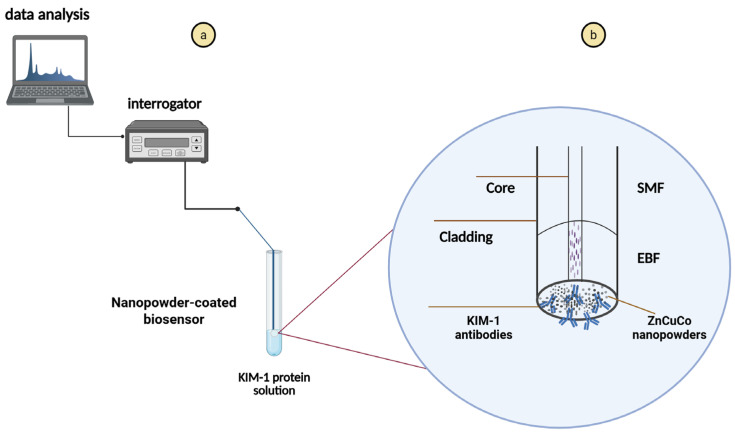
(**a**) A schematic representation of an experimental arrangement designed for real-time biomarker analysis and (**b**) zoom out of SMF—EBF optical fiber biosensor functionalized with hydrothermal-synthesized ZnCuCo_2_O_4_ (No. 1 sample) NPs and KIM-1 antibodies. Created in BioRender. Balgimbayeva, U. (2025) https://BioRender.com/aobhjxi (accessed on 21 April 2025).

Figure 3b demonstrates an experimental overview of measuring the KIM-1 biomarker using the biofunctionalized optical fiber biosensor coated with nanopowders. To detect protein levels using the functionalized biosensors, a series of recombinant KIM-1 protein dilutions were prepared in PBS, spanning concentrations from 1 aM to 100 nM. In total, twelve concentrations of protein were prepared from 100 nM to 1aM, and seven of these concentrations were used (100 nM, 10 nM, 100 pM, 1 pM, 10 fM, 100 aM, and 1 aM) for detection. The measurements started with a blank solution as a control. For each concentration, experiments were conducted by taking 10 readings, at one-minute intervals under stable room temperature (25 °C) conditions to reduce potential interferences.

The interrogation of the SDI sensors was performed using a dynamic FBG interrogator (Micron Optics si255, Roanoke, VA, USA) operating in spectral scanning mode. This interrogator integrates a swept laser source and an array of photodetectors, with each sensing channel physically separated for parallel analysis. Briefly, the sensor operates entirely based on the properties of the EBF, which is spliced to a SMF. The EBF contains intrinsic scattering centers that function as multiple partially reflective interfaces, enabling a distributed interferometric response. At the distal end, the cleaved tip of the EBF creates a Fresnel reflection at the interface with the protein solution, forming an RI-dependent reflective surface. On the proximal side, the EBF exhibits an increased Rayleigh scattering profile compared to a standard SMF, which provides the necessary reflection to establish a cavity. Along the length of the EBF, the continuous distribution of scattering centers forms intermediate reflection layers that modulate the spectral response. When light from the interrogator is launched into the SMF, it propagates through the fiber and enters the EBF region. Inside the EBF, the light experiences multiple distributed back-reflections due to the microstructural inhomogeneities, effectively forming a Fabry–Perot cavity. These reflections interfere coherently, generating a measurable interferometric signal that shifts in response to changes in the surrounding refractive index caused by protein binding.

## 3. Results and Discussion

### 3.1. Characterization of Copper, Zinc, and Cobalt Metal Oxide Nanopowders

**Figure 4:**
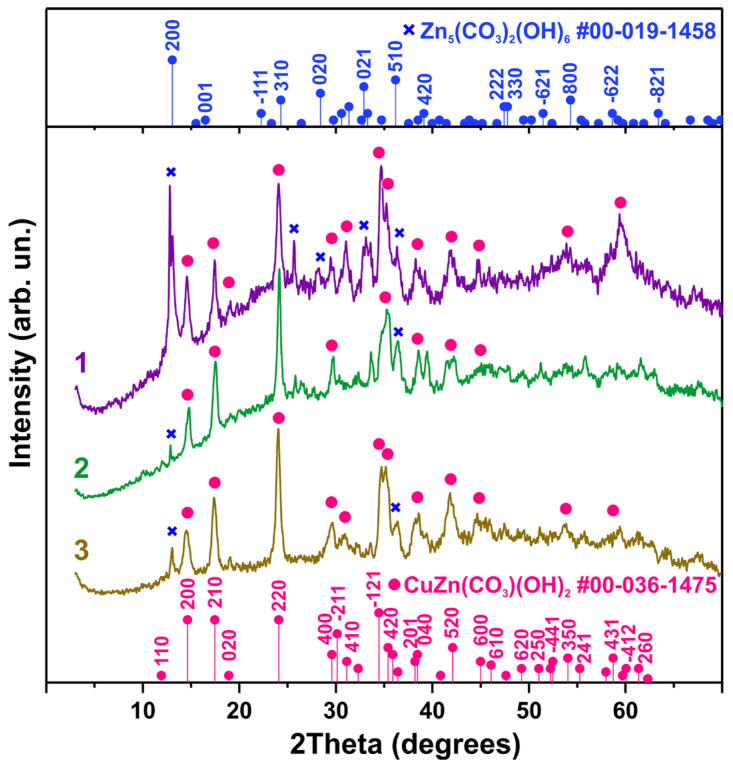
Results of XRD analysis of synthesized samples.

Synthesized nanopowder was characterized using several analytical methods. It should be noted that despite the differences in the compositions of growth solutions during the synthesis of the samples, the results of XRD, SEM, and Raman spectroscopy of all three samples were very similar. As can be seen from Figure 4, the XRD results of all the samples are close, and the positions of the major reflections are the same for all three samples. All the observed XRD reflections can be described by the presence of two hydroxycarbonate phases in the samples, namely, first, CuZn(Co_3_)(OH)_2_, corresponding to JCPDS map No. 01-036-1475, and Zn_5_(CO_3_)_2_(OH)_6_, this phase corresponds to JCPDS map No. 00-019-1458. The width of the XRD peaks in all three diffractograms is similar and significant, as can be seen in Figure 4. From the six single reflections of sample No. 3, the average crystallite size was estimated using Scherrer’s formula [19], which was found to be 9.5 ± 1.8 nm. It should be noted that the XRD results and morphology of the samples did not change after annealing at 200 °C for 5 h in air, which indicates the stability of the structure.

**Figure 5:**
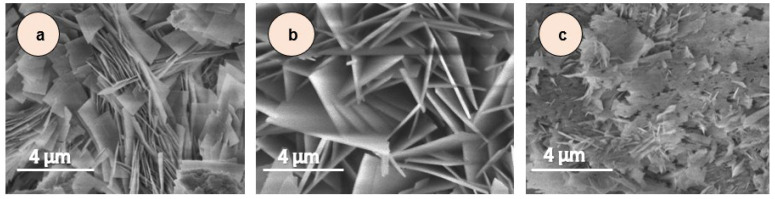
SEM images of samples of ZnCuCo_2_O_4_ NPs in different molar ratio precursors of Co:Zn:Cu, (**a**) No. 1 (4:1.8:0.2), (**b**) No. 2 (4:0.2:1.8), and (**c**) No. 3 (4:1:1).

Figure 5 shows SEM images of samples No. 1–No. 3. The morphology of the samples is approximately the same and consists of thin plates with a thickness of about 40 nm.

**Figure 6:**
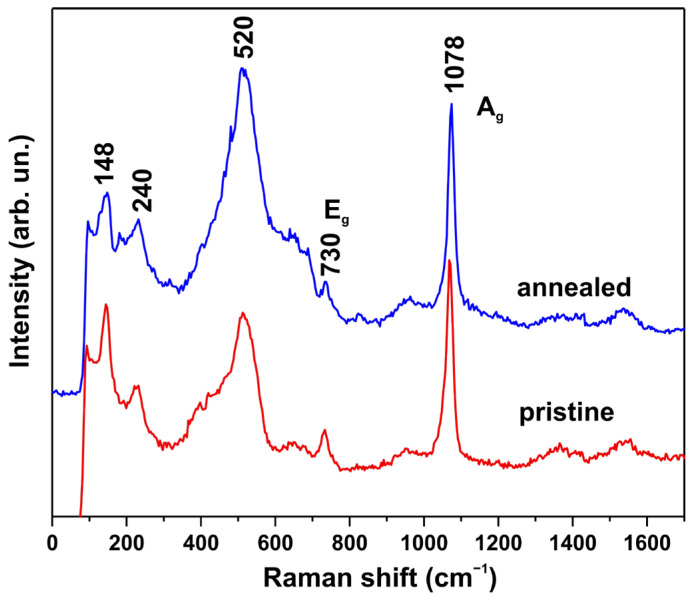
Raman spectra of sample No. 1 before and after annealing at 200 °C in air.

Figure 6 shows the Raman spectra of sample No. 1 before and after annealing at 200 °C. Other samples showed similar spectra. On the spectra, the presence of an intense line at 1078 cm^−1^ and a weak but narrow band at 730 cm^−1^ indicates the presence of Me-(CO_3_) groups in metal carbonates [23], in which metal substitution causes only a weak line shift [25]. The identification of the low-frequency lines at 520, 240, and 148 cm^−1^ is not as obvious, but they can also be attributed to the structure of metal carbonates, which show a number of lines in this region [23]. Also, as in the case of XRD and SEM studies, the Raman spectra do not show noticeable changes after annealing, which proves the relative thermal stability of the obtained hydroxycarbonates.

### 3.2. Surface Analysis After Nanopowder Coating

**Figure 7:**
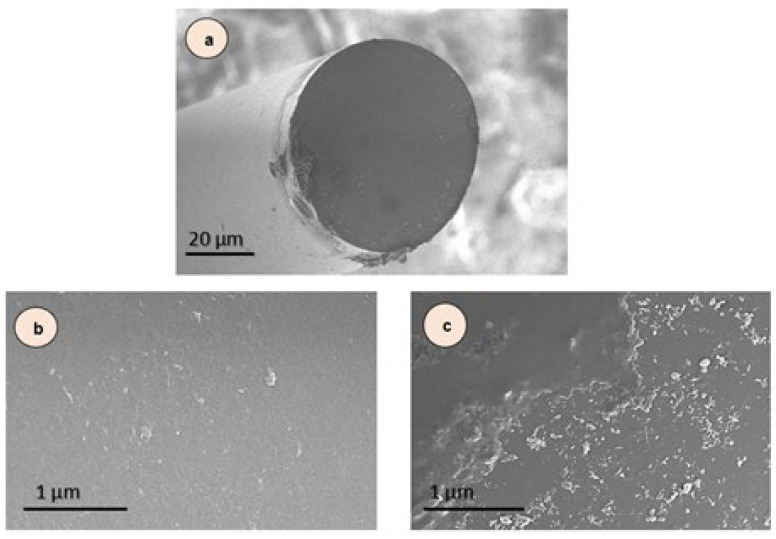
SEM images of the optical fiber sensor before (**a**,**b**) and after coating with nanopowder (**c**), (**a**) and (**b**) after Piranha solution 497× (**a**) and 5000× magnification (**b**), and (**c**) after ZnCuCo_2_O_4_ (No. 1 sample) nanopowder layer coating.

Figure 7 shows SEM images of optical fibers of different types. In Figure 7a, the sensor after the Piranha solution shows a clean sensor surface free from impurities. Also, Figure 7b shows a zoom out of the sensor without NPs coating. Figure 7c shows the sensor after the ZnCuCo_2_O_4_ (No. 1 sample) NPs layer coating. The results of the electron microscopy study showed that the ZnCuCo_2_O_4_ (No. 1 sample) NPs layer applied in fine particles over the entire surface area of the sensor. Thus, the immersion dip-coating method allows coating nanoparticles on the sensor to bind the main components of ZnCuCo_2_O_4_ to other organic matter, such as Piranha solution, MUA, KIM-1 antibodies, and BSA solution.

### 3.3. Spectral Analysis and Refractive Index Detection

The RI plays a critical role in determining the performance of an optical fiber interferometer. Each spectral peak and valley within the interferometer’s output exhibits sensitivity to changes in RI, with an increase in RI reducing the mirror reflectivity at the fiber’s tip. Sensitivity, however, varies depending on the specific spectral feature being analyzed. For instance, valleys, influenced by destructive interference, tend to show more pronounced intensity changes in response to RI variations. The robustness of the sensor’s performance is reflected in the high consistency of measurements, with R^2^ values exceeding 0.95 across all spectral features. On average, the sensitivity measures approximately 1 dB/RIU, with a standard deviation of 58 dB/RIU, aligning closely with the performance of SDI/FBG probes.

**Figure 8:**
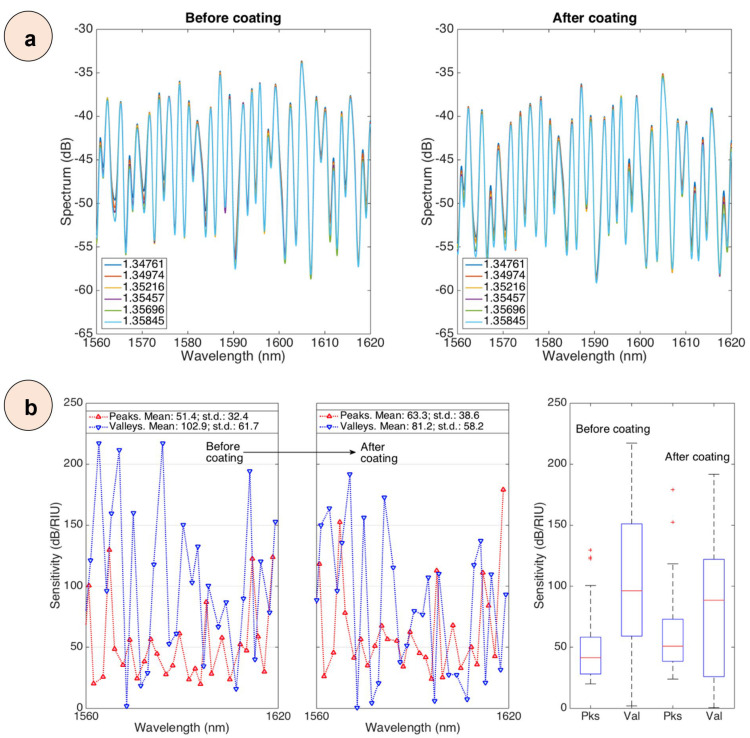
(**a**) Spectra of an SDI sensor for various RI values (from 1.34761 to 1.35845), acquired before (**left**) and after (**right**) the nanopowder coating. (**b**) Refractive index sensitivity for an SDI sensor. (**Left**): sensitivity (absolute value) reported for each spectral feature identified in the spectrum before and after the nanopowder coating. (**Right**): Boxplot of the refractive index sensitivity for peaks and valleys in the SDI spectrum before and after the coating.

The FBG spectrum remained unchanged throughout the experiment, which corresponds to the existing theory that Bragg gratings located in the fiber core react weakly to changes in the RI of the environment. FBG has a linear sensitivity to temperature and, due to its independence from RI, can serve as a reliable reference for accurate monitoring of temperature changes [10]. Therefore, our analysis focused on the spectral range from 1560 to 1620 nm. Figure 8 shows the spectra of the SDI sensor, which give an idea of the spectrum of the device before and after nanopowder coating and detection after functionalization (smallest and highest protein concentrations). The return loss ranged from −58 dB to −33 dB, which indicates reliable signal transmission characteristics. The application of nanopowder coatings led to significant changes in spectral properties, as evidenced by a clear shift in the spectrum by about −3 dB between uncoated and coated states. They demonstrate both changes in spectral properties, which depend on the concentration, and changes caused by the coating itself.

To study the sensitivity characteristics of RI, a set of spectral measurements were carried out, the results of which are shown in Figure 8a. To quantify the spectral response, a reliable peak tracking algorithm was developed that tracked intensity changes for individual modes depending on the frequency range. The algorithm used the spline interpolation method to accurately identify and localize these features. The intensity values at the identified spectral positions were then extracted and used to calculate the sensor response in decibels (dB) [11]. To more accurately characterize these intensity modulations, a second-order polynomial fit was used, which allowed accurate sensitivity measurements over the entire spectral range. A comparative analysis of the spectral characteristics before and after the coating showed that the fundamental interference pattern was preserved. This indicates that the nanopowder coating did not affect the basic mechanisms of modal interference. However, a more detailed study revealed subtle but obvious changes in the position of the peaks and the intensity distribution. These changes can be seen in the form of wavelength shifts and amplitude changes in response to RI changes. This suggests that the nanopowder coating effectively modulates the interaction between the guided modes and the external medium, altering the interferometric signal. The changed sensitivity profile of RI after coating indicates that the coating of the sensor surface was successful. Spectral characteristics, including peaks and valleys with an intensity of more than 1.5 dB, were identified using the envelope shown in Figure S1. At the same time, the RI sensitivity of the SDI sensors was calculated by analyzing changes in the intensity of spectral peaks and valleys, as shown in Figure S2.

**Figure 9:**
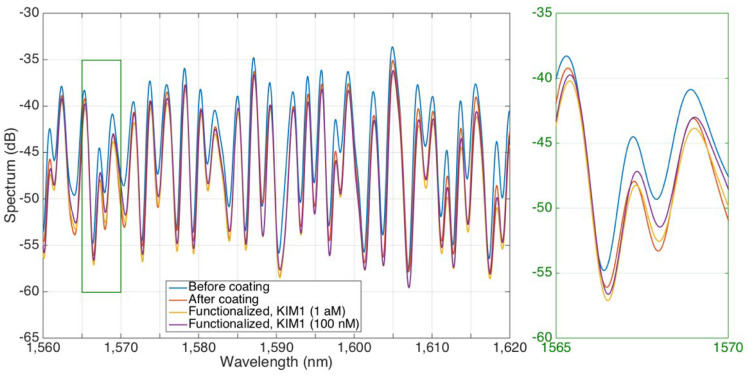
Spectra of an SDI sensor through the steps of functionalization and detection; the spectrum is reported before and after the coating with nanopowder, and during KIM-1 detection (lowest and highest concentrations). The inset on the right shows a 5 nm spectral window.

Sensitivity analysis of uncoated sensors showed that spectral valleys reach a maximum sensitivity of 220 dB/RIU, with an average value of 102.9 dB/RIU and a standard deviation of 61.7 dB/RIU. In contrast, spectral peaks have lower sensitivity: the maximum, average, and standard deviations are 130 dB/RIU, 51.4 dB/RIU, and 32.4 dB/RIU, respectively. These results are consistent with data from previous studies, which also showed higher sensitivity in spectral valleys compared to peaks [10]. After the coating, the analysis revealed changes in sensitivity characteristics. Spectral valleys demonstrated a maximum sensitivity of 190 dB/RIU, an average of 81.2 dB/RIU, and a standard deviation of 58.2 dB/RIU. The corresponding spectral peaks showed values of 180 dB/RIU, 63.3 dB/RIU, and 38.6 dB/RIU (Figure 8b, left). The wavelength shift analysis showed ranges from 18 dB/RIU to 102 dB/RIU for spectral peaks and from 3 dB/RIU to 9.391 dB/RIU for spectral troughs. The average values are 6.004 dB/RIU and 7.232 dB/RIU, respectively (Figure 8b, right). Statistical analysis confirmed the high reliability of the system. All spectral characteristics demonstrated a coefficient of determination R^2^ exceeding 0.95. The RI detection accuracy was approximately 10^−4^ RIU, and the noise level of the analyzer was less than 0.05 dB. Figure 9 shows the spectrum of one sensor, through the coating, functionalization, and detection steps, and an inset to visualize the changes occurring in each spectral feature.

The dip-coating method is a straightforward and cost-effective approach for applying nanopowder coatings to optical fiber sensors [29]. It ensures deposition of metal oxides, such as zinc, copper, and cobalt, onto the fiber surface, enhancing the sensor sensitivity and stability.

Sensitivity is measured in mV/ppm (millivolts per part per million), indicating the sensor’s response per unit change in RI or analyte concentration [30]. In this case, highlight the significant impact of nanoparticle material and sensor configuration on the sensitivity of optical fiber sensors. Copper-coated optical fibers have demonstrated high sensitivity in RI sensing, while gold and silver nanoparticles on D-type and tapered optical fibers show varying sensitivities in detecting volatile liquids [15]. The choice of nanoparticle material and fiber geometry are crucial factors in optimizing sensor performance for specific applications [30].

### 3.4. Measuring the Stability

**Figure 10:**
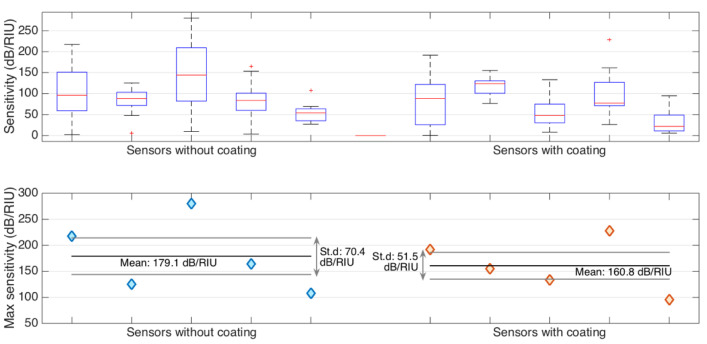
Comparison of refractive index sensitivity of 10 SDI sensors, 5 without coating and 5 with nanopowder coating. (**Upper chart**): boxplot showing the refractive index sensitivity of the spectral valleys, evaluated for each sensor. (**Lower chart**): maximum sensitivity, displaying the maximum value for each sensor, and the average/standard deviation for each set of 5 sensors.

A study of the effect of the nanopowder coating on sensor operation demonstrated changes in both sensitivity and measurement stability. These changes are clearly shown in Figure 10. From the upper panel graph below, we can see that uncoated sensors showed a high degree of sensitivity variability during the measurement process. In some cases, the peak sensitivity reached more than 200 dB/RIU. Although this high variability can be useful for some highly sensitive applications, it also poses problems for reproducibility of measurements and reliability of the sensor. Unlike uncoated sensors, nanopowder-coated sensors have demonstrated more stable performance with lower dispersion. The lower part of the graph shows quantitative data confirming the compromise between maximum sensitivity and measurement stability. Uncoated sensors showed an average maximum sensitivity of 179.1 ± 70.4 dB/RIU. However, a large standard deviation indicates significant fluctuations in the sensor response. For comparison, nanopowder-coated sensors demonstrated a slightly lower but more stable sensitivity of 160.8 ± 51.5 dB/RIU. The reduction of the standard deviation by about 27% indicates a significant improvement in measurement reliability. This trade-off between sensitivity and reproducibility is an interesting contrast to the recent study of Ashikbayeva et al. (2023) [28]. In their study, they demonstrated that the use of gold nanoparticles obtained using environmentally friendly methods made it possible to increase the sensitivity of fiber-optic biosensors for detecting cancer biomarkers by 25 times. We deliberately chose more economical nanopowder instead of expensive gold nanoparticles. Our goal was to make measurements cost-effective and consistent, rather than striving for maximum sensitivity. This compromise is better suited to the practical requirements of on-site diagnosis and routine clinical monitoring. Although the surface coating process reduced the maximum sensitivity by 10.2%, it significantly improved the reproducibility of measurements. This has important practical implications for real-world sensing applications where reliability and stability play a key role.

### 3.5. Detection of KIM-1 Protein Biomarker

**Figure 11:**
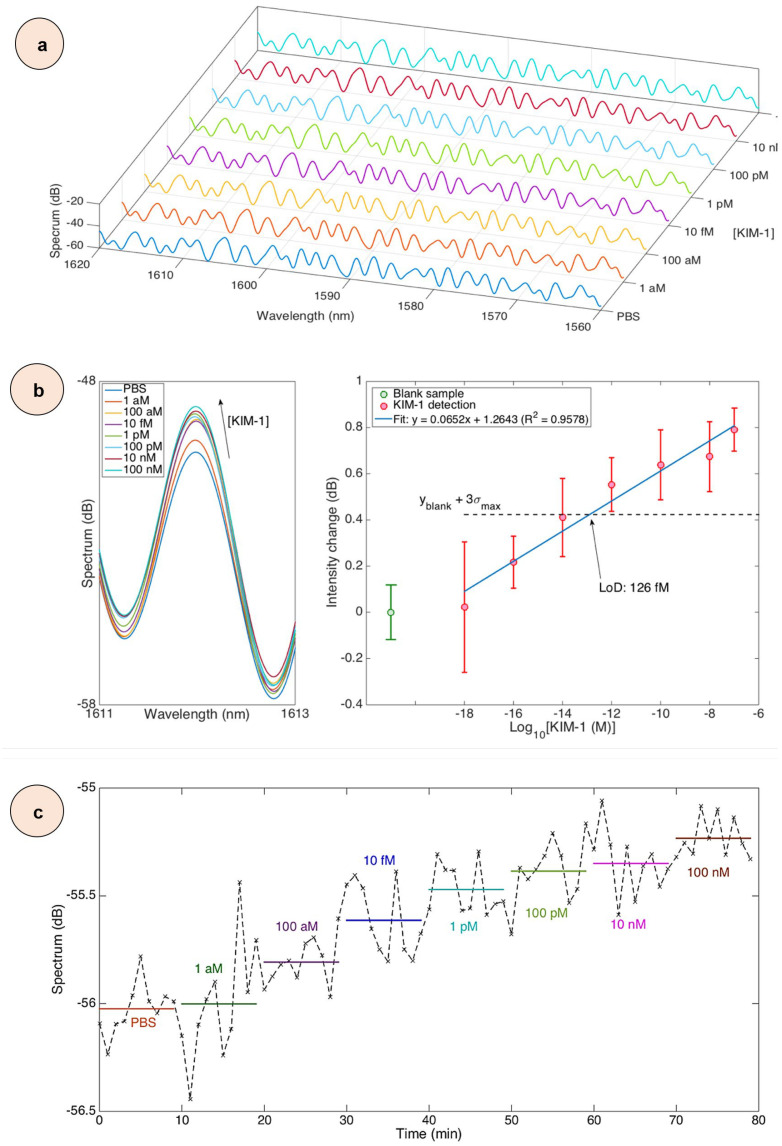
(**a**) Spectra of a KIM-1 functionalized SDI biosensor (coated with ZnCuCo_2_O_4_ nanopowders (No. 1 sample)) after 5 min exposure to concentrations of KIM-1 from 1 aM to 100 nM; (**b**) Left: spectral change observed for a spectral feature corresponding to a peak at 1612 nm with increasing KIM-1 concentration. Right: response of the SDI to KIM-1 concentration; error bars display mean value ± standard deviation of 7 consecutive measurements; and (**c**) sensorgram of the SDI biosensor, reporting the kinetic response at various KIM-1 concentrations with 1 min sampling time.

In order to perform a comprehensive detection analysis of the KIM-1 protein using an optical fiber SDI biosensor coated with nanopowders, we have focused on its detection capabilities and performance characteristics over a wide range of concentrations. The evaluation was carried out in a PBS buffer, which made it possible to establish the basic parameters of the sensor and minimize possible interference from other molecules. Spectral analysis showed different modulation patterns during KIM-1 binding, as can be seen in Figure 11a. These patterns were characterized by wavelength shifts in the interference fringes, which directly correlated with protein concentration. It is possible to see how the spectral characteristics change, which indicates that the biomolecular detection was successful. These changes indicate a close interaction between the sensor surface and KIM-1 proteins, and at higher concentrations, responses become more pronounced.

A thorough analysis of the spectral range from 1611 to 1613 nm, shown in Figure 11b, focuses on the characteristic peak at 1612 nm. This study revealed clear shifts that depend on protein concentration. The left panel of Figure 11b shows these spectral changes, and the right panel shows a quantitative assessment of the reaction at various concentrations of KIM-1. Our fabricated sensor demonstrated high stability, maintaining a baseline standard deviation of ±0.1 dB for eight consecutive measurements. In addition, by selecting the most sensitive function, we can evaluate the sensor response, as shown in Figure 11b (right). Moreover, the concentration-to-response ratio demonstrates a systematic progression spanning several orders of magnitude. Even at the lowest tested concentration of 1 aM, the sensor showed a detectable response of approximately 0.05 dB. The response value gradually increased with increasing concentration, reaching 0.18 dB at 100 aM, 0.34 dB at 10 fM, 0.46 dB at 1 pM, 0.55 dB at 100 pM, 0.68 dB at 10 nM, and finally 0.81 dB at 100 nM. The maximum standard deviation observed in all measurements was ±0.24 dB, which indicates acceptable signal variability even at higher concentrations. Real-time kinetic analysis, shown in the sensorgram in Figure 11c, showed concentration-dependent binding dynamics when sampling data with an interval of 1 min. Stable and reliable responses were observed at higher concentrations (10–100 nM), while at lower concentrations (1–100 aM) the signal changes were more subtle and the baseline level was less stable. This pattern demonstrates a complex interaction between the binding rate and signal stability in different concentration ranges. Interestingly, this interaction can be clearly traced in the temporal evolution of the sensor response. Furthermore, analysis of the concentration response using linear regression showed that with a 10-fold increase in the concentration of KIM-1, the response increases by 0.0652 dB, with a strong correlation coefficient (R^2^ = 0.96). The analytical detection limit (LoD), calculated using the formula $x_{LoD}=f^{-1}\left(y_{blank}+3\sigma_{max}\right)$ (y*_blank_* = blank sample level; σ*_max_* = maximum standard deviation) [31], is 126 fM.

### 3.6. Specificity

**Figure 12:**
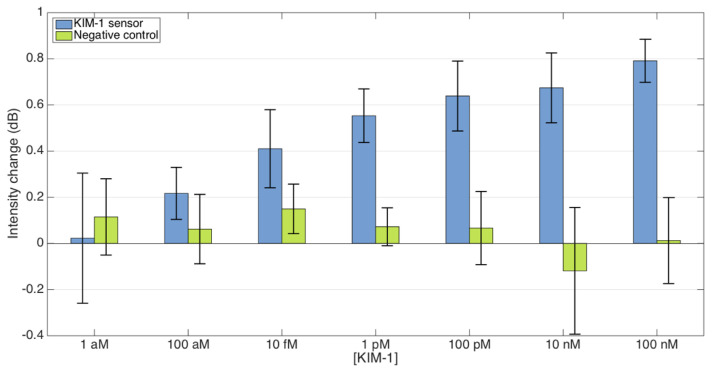
Specificity analysis, comparing the biosensor functionalized with KIM-1 antibody and a negative control for various concentrations.

For the specificity studies, we conducted a comparative analysis of biosensor performance by analyzing the signal change as the concentration of KIM-1 increases using a control biosensor without the antibodies (negative control). Figure 12 shows the measurement results that showed how the intensity of the sensor response decreases compared to the baseline level at various concentrations of the KIM-1 protein (from 1 aM to 100 nM) in a PBS buffer solution. As a result, the KIM-1-antibody-functionalized biosensors exhibited a clear concentration-dependent response pattern. Interestingly, at 100 nM, we observed maximum signal intensity changes of approximately 0.85 dB. Even at extremely low concentrations (1 aM), the biosensor maintained detectable signals (~0.18 dB) that were distinguishable from the negative control. Moreover, intermediate concentrations of 1 pM and 10 pM produced intensity changes of approximately 0.45 dB and 0.65 dB, respectively. If we now turn to the negative control sensors, fabricated without antibodies, they maintained baseline readings (~0 dB) throughout the concentration range, confirming the specificity of our KIM-1 detection system.

### 3.7. Reproducibility

A detailed reproducibility analysis was performed using three different SDI biosensors coated with ZnCuCo_2_O_4_ (No. 1, 2, and 3 samples) nanopowders that responded to the concentration of the KIM-1 protein in the range from 1 aM to 100 nM. It can be seen from the data in Figure S3 that each sensor had its own response characteristics, but the detection principle remained unchanged over the entire concentration range. A quantitative analysis of sensor responses, shown in Figure S3a, demonstrated different sensitivity profiles for each of them. Sensor 2 showed the highest sensitivity, with a slope of 0.1461 dB for each 10-fold increase in concentration. At the same time, sensors 1 and 3 showed more moderate responses with slopes of 0.0652 dB and 0.0420 dB for each 10-fold increase in concentration, respectively. The range of sensitivity changes that we observed demonstrates that even minor modifications during the manufacturing and surface treatment process can significantly affect the final performance of each sensor. In addition, we noted a difference in the maximum sensitivity of the sensors. At the maximum concentration that was tested (100 nM), Sensor 2 showed exceptional sensitivity, varying the intensity by about 1.6 dB. Meantime, Sensors 1 and 3 showed lower but stable maximum responses, varying the intensity by 0.9 dB and 0.8 dB, respectively. The differences in the maximum output signal that can be observed from different sensors are probably related to the characteristics of the active detection surface and the features of optical transmission. Figure 11b shows a normalized response analysis that highlights key aspects of sensor operation over a wide range of concentrations. All three sensors demonstrated a stable linear response in the concentration range from 1 aM to 10 pM, which indicates the high reliability of their detection in this area. With increasing concentration, a characteristic reaction was observed: all sensors showed a slight decrease in the signal at 10 nM before reaching its maximum value at 100 nM. This stable behavior pattern observed for all three sensors, despite their different sensitivities, indicates a fundamental mechanism of interaction between the sensor surface and the KIM-1 protein at higher concentrations. Although the sensors have different absolute sensitivity, their sequence of reactions indicates the reliability of the detection mechanism and the reproducibility of the basic principle of operation in various sensor units.

## 4. Conclusions

In this article, we present an optical fiber biosensor platform based on an SDI coated with Zn, Cu, and Co metal oxide nanopowders to detect the KIM-1 protein biomarker. The sensor’s design is simple to fabricate, which makes its production not only fast but also economically profitable. In addition, the use of a nanopowder coating makes it possible to improve the sensor’s performance while maintaining its simplicity and increasing the reproducibility of measurements. The biosensor has shown impressive results in terms of sensitivity. The average sensitivity of the coated sensors was 160.8 ± 51.5 dB/RIU, which is comparable to modern optical biosensor platforms. Moreover, in the course of our systematic research, we have identified unique detection capabilities for KIM-1 with the theoretical detection limit of 126 fM, and the experimental detection capability reached the attomolar range (1 aM). The wide dynamic range of the system, which covers from 1 aM to 100 nM, is particularly impressive. This is a significant step forward in the development of biosensor technologies. Comprehensive specificity checks and reproducibility analyses confirm the reliability and practical usefulness of the sensor. The nanopowder coating strategy not only ensures the consistency of measurements but also contributes to the stability of the biosensor, which demonstrates the potential of the platform for reliable detection of biomarkers in complex biological matrices. The results obtained using several sensors indicate high measurement accuracy. The maximum standard deviation is only ± 0.24 dB, which indicates the reliability of our approach.

The sensitivity and performance of this sensor system can be significantly strengthened through the use of innovative fiber designs and nanotechnology. Tapered optical fibers and fibers with a D-shaped profile or polished on the sides can significantly increase the interaction with the biomolecules targeted by the sensor. Meantime, specialized architectures, such as photonic crystal fibers, open up additional possibilities for optimizing the interaction of light with matter. Post-processing techniques such as surface etching, plasmonic coatings of nanoparticles, and localized amplification of surface plasmonic resonance can significantly increase sensor sensitivity, potentially reducing detection limits below the current attomolar range. This will allow us to create optimized sensor options for various applications based on machine learning algorithms.

The proposed sensor is characterized by simplicity of manufacture, high sensitivity, and good reproducibility. Due to these qualities, it is perfectly suited for real-time on-site diagnostics and continuous monitoring of biomarkers. The sensor has femtomolar sensitivity, which makes it ideal for single-use applications in clinical settings where cost-effectiveness is important. The interferometric setup provides accurate real-time detection, surpassing traditional intensity-based methods. Overall, this sensor is characterized by reliability, reproducibility, and high sensitivity, making it an excellent choice for clinical diagnostics and medical applications.

## Data Availability

Data are available upon request.

## Supplementary Materials

The following supporting information can be downloaded at: https://www.mdpi.com/article/10.3390/bios15070449/s1, Figure S1: Envelope used to evaluate all the peaks/valleys of the SDI spectrum with peak prominence > 1.5 dB; Figure S2: Method for calculation of the RI sensitivity for SDI sensors. The chart shows, for each spectral feature identified in the envelope of the spectrum (red = peaks; blue = valleys), the intensity (mean st. dev. of 3 measurements) and the linear regression to estimate the RI sensitivity; Figure S3: (a) Reproducibility analysis, showing the response and the sensitivity of 3 biosensors undergoing the same functionalization and measurement of KIM-1 in PBS buffer; (b) Reproducibility analysis, showing the normalized response of three biosensors for various concentrations of KIM-1 protein. The response is normalized (0 = PBS; 1 = KIM-1 max. concentration, 100 nM). Colored lines = mean responses of each sensor; black line: mean value of the response; shadowed region: standard deviation).
