# Supplementary material for: Composite Metal Oxide Nanopowder-Based Fiber-Optic Fabry–Perot Interferometer for Protein Biomarker Detection
Source: Biosensors (Basel). 2025 Jul 13;15(7):449. doi: 10.3390/bios15070449 (PMC12293339; doi:10.3390/bios15070449)
Supplement: Supplementary file 1 [file biosensors-15-00449-s001.zip › biosensors-3636254-supplementary.pdf]

biosensors

MDPI

Supplementary

Composite Metal Oxide Nanopowder-Based Fiber-Optic Fabry–Perot Interferometer for Protein Biomarker Detection

Ulpan Balgimbayeva [1], Zhanar Kalkozova [2], Kuanysh Seitkamal [3], Daniele Tosi [3,4,*], Khabibulla Abdullin [1,2,*] and Wilfried Blanc [5]

[1] School of Materials Science and Green Technology, Kazakh-British Technical University, Almaty 050000, Kazakhstan; u.balgimbaeva@kbtu.kz
[2] National Nanotechnology Laboratory of Open Type, Al-Farabi Kazakh National University, Almaty 050040, Kazakhstan; zhanar.kalkozova@kaznu.edu.kz
[3] Laboratory of Biosensors and Bioinstruments, National Laboratory Astana, Nazarbayev University, Astana, 010000 Kazakhstan; kuanysh.seitkamal@nu.edu.kz
[4] School of Engineering and Digital Sciences, Nazarbayev University, Astana, 010000 Kazakhstan
[5] INPHYNI, CNRS UMR7010, Université Côte d'Azur, 17 rue Julien Lauprêtre, 06200 Nice, France; wilfried.blanc@inphyni.cnrs.fr
* Correspondence: daniele.tosi@nu.edu.kz (D.T.); kh.abdullin@physics.kz (K.A.)

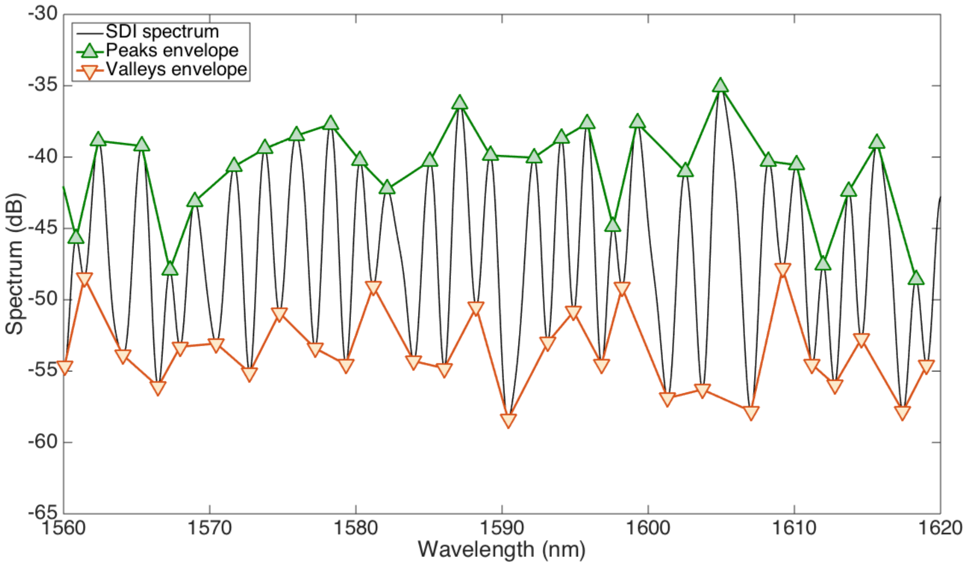

Figure S1. Envelope used to evaluate all the peaks/valleys of the SDI spectrum with peak prominence > 1.5 dB.

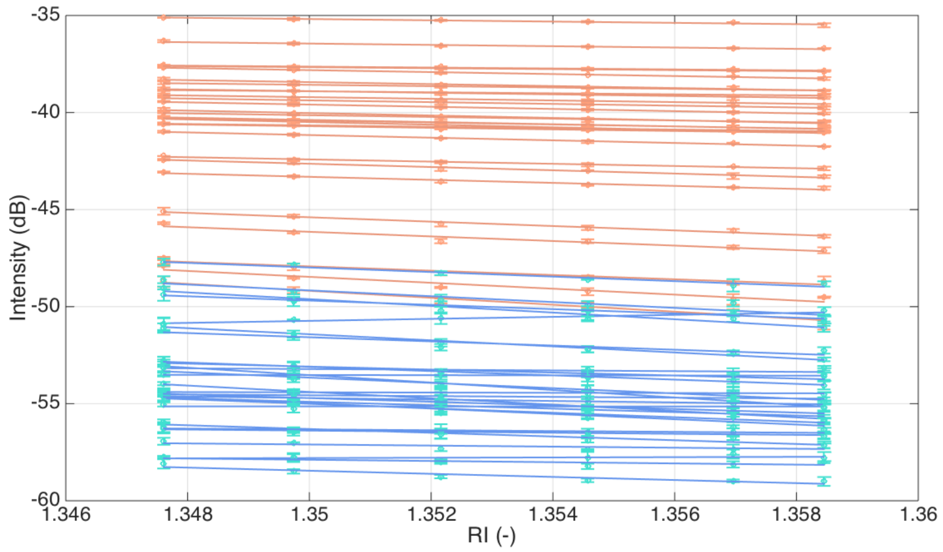

Figure S2. Method for calculation of the RI sensitivity for SDI sensors. The chart shows, for each spectral feature identified in the envelope of the spectrum (red = peaks; blue = valleys), the intensity (mean st. dev. of 3 measurements) and the linear regression to estimate the RI sensitivity.

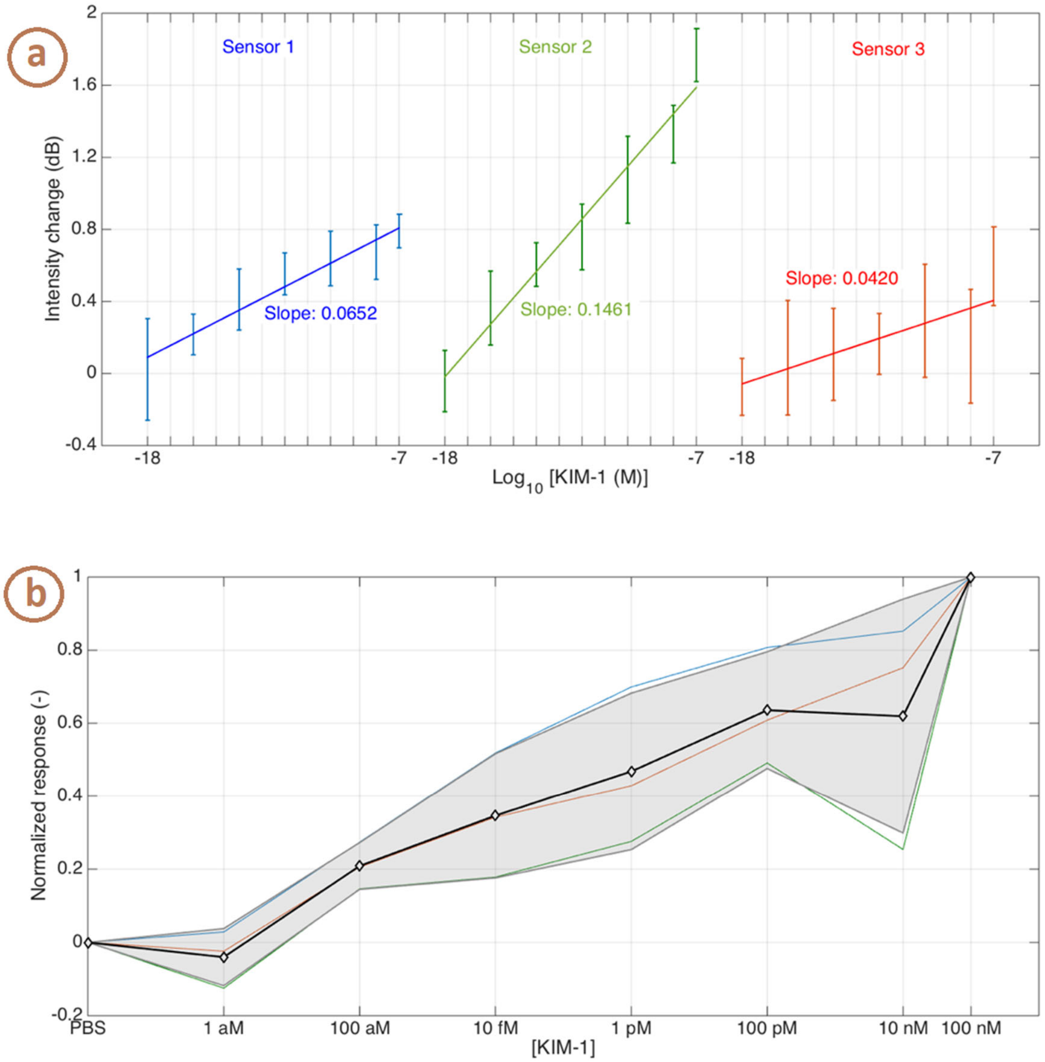

Figure S3. (a) Reproducibility analysis, showing the response and the sensitivity of 3 biosensors undergoing the same functionalization and measurement of KIM-1 in PBS buffer; **(b)** Reproducibility analysis, showing the normalized response of three biosensors for various concentrations of KIM-1 protein. The response is normalized (0 = PBS; 1 = KIM-1 max. concentration, 100 nM). Colored lines = mean responses of each sensor; black line: mean value of the response; shadowed region: standard deviation).
